# Granulomatosis With Polyangiitis Mimicking Infective Endocarditis: A Case Report

**DOI:** 10.7759/cureus.70844

**Published:** 2024-10-04

**Authors:** Yusra Ansari, Saad Ali Ansari, Fawaz Mohammed, Zaina Ali Khan, Zunaira Ansari, Dylan Sanford, Tahir Muhammad Abdullah Khan

**Affiliations:** 1 Internal Medicine, University of Kentucky College of Medicine, Bowling Green, USA; 2 Internal Medicine, University of California Riverside School of Medicine, Riverside, USA; 3 College of Medicine, Deccan College of Medical Sciences, Hyderabad, IND; 4 Medical Education, University of the Witwatersrand, Johannesburg, Johannesburg, ZAF; 5 Pulmonary and Critical Care Medicine, University of Kentucky College of Medicine, Bowling Green, USA; 6 Internal Medicine, Marshfield Medical Center, Marshfield, USA

**Keywords:** granulomatosis with polyangiitis (gpa), infective endocarditis, leukoclastic vasculitis, recurrent sinusitis, wegners granulamatosis

## Abstract

Granulomatosis with polyangiitis is a rare systemic disease that causes necrotizing granulomatous inflammation of small- and medium-sized blood vessels. We present the case of a 46-year-old male with medical history significant for chronic sinusitis, prior history of drug abuse, and a recent tooth infection. He was suspected to have infective endocarditis, but further workup revealed diagnostic findings of granulomatosis with polyangiitis. We discuss how the signs and symptoms of granulomatosis with polyangiitis can overlap with infective endocarditis, a pathophysiologically distinct condition with a strikingly similar presentation.

## Introduction

Granulomatosis with polyangiitis (GPA), previously known as Wegener granulomatosis, is a necrotizing vasculitis of the small and medium-sized vessels. The incidence of GPA in the United States is approximately 3 per million, and it is generally seen in older age groups, with recent studies showing it is equally distributed among both males and females [1]. The etiology of GPA is not well understood. However, the etiopathogenesis has been proposed to be due to anti-neutrophilic cytoplasmic antibodies (ANCA), presumed to be the cause of inflammation in GPA. Cytoplasmic ANCA (c-ANCA), where autoantibodies are directed against proteinase 3 antibodies, is seen in up to 90% of GPA cases, whereas perinuclear ANCA (p-ANCA), directed against myeloperoxidase antibodies, is seen in only a handful of cases. ANCAs targeted against proteinase 3 (PR3-ANCA) are highly specific for GPA [2]. We report an interesting case of a 46-year-old male whose presentation and history were suggestive of infective endocarditis (IE) but who was ultimately diagnosed with GPA.

## Case presentation

**Figure 1:**
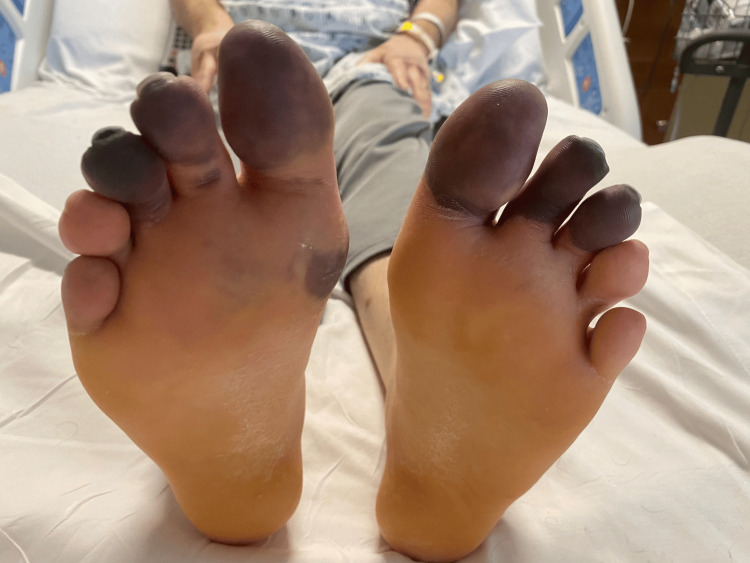
Bilateral toes showing bluish discoloration secondary to vasculitis of the involved vessels

A 46-year-old male with history of nasal congestion and failure of outpatient antibiotics for presumed sinusitis presented to an outside facility with worsening sinus pressure. He reported chills but denied having fevers, although he noted he was taking acetaminophen and ibuprofen at the time. He also reported pain at the level of his toes with black and blue discoloration. His past medical history was significant for anxiety, post-traumatic stress disorder, and gastroesophageal reflux disease. Physical examination revealed purplish discoloration of the bilateral toes, which were painful to touch (Figure 1).

**Figure 2:**
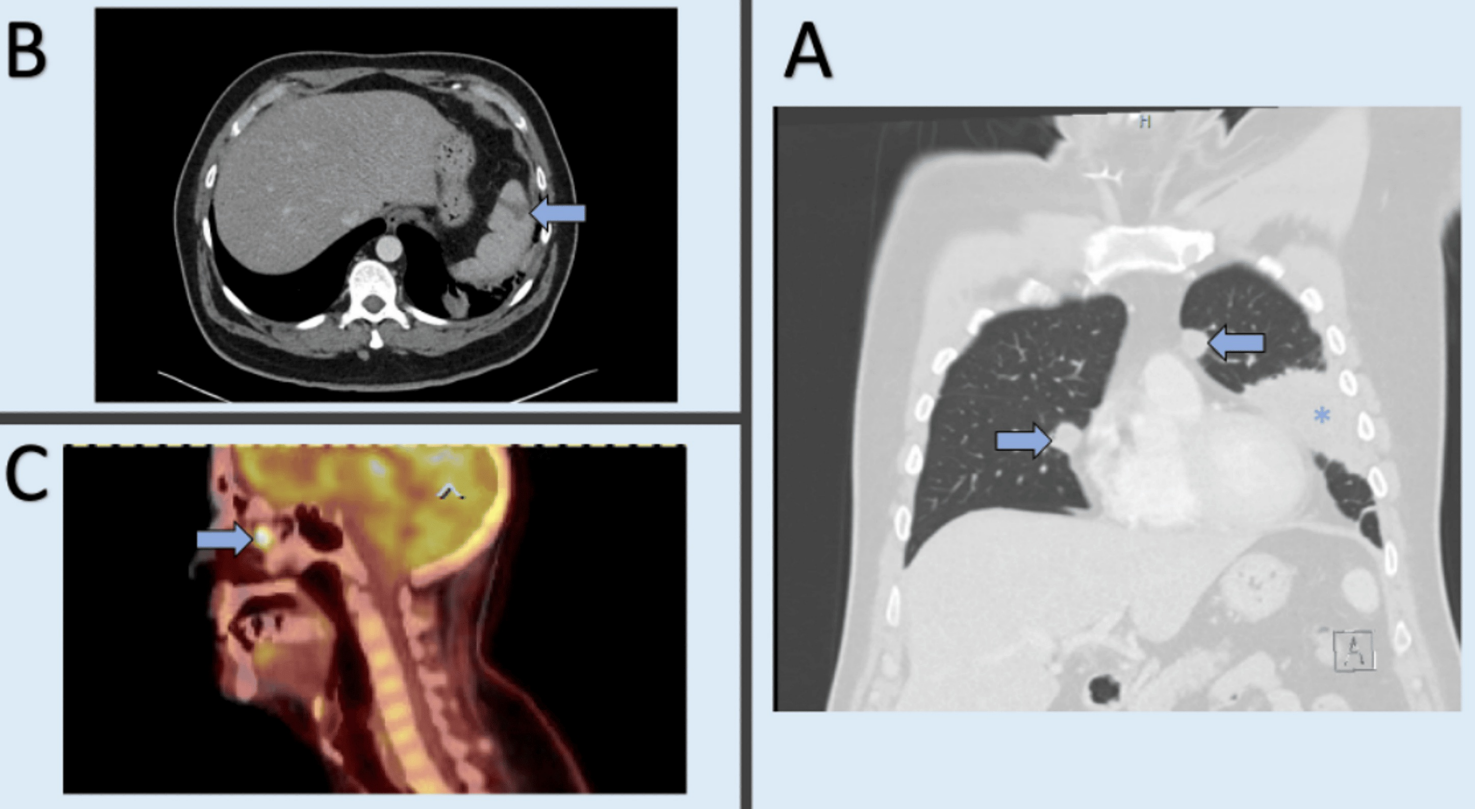
Imaging findings (A) CT chest showing a nodular cavitary process in the lung fields (arrows) and lingular consolidation (asterisk); (B) wedge-shaped hypo-perfusion within the spleen, consistent with splenic infarction (arrow); (C) PET scan showing abnormal increased activity in the nasal area (arrow).

Liver function tests, creatinine, and lactic acid levels were within normal limits. Significant laboratory findings are tabulated in Table 1. Urinalysis showed 10-25 white blood cells, with negative nitrites. A computed tomography (CT) scan of the chest was obtained for further evaluation, revealing evidence of a splenic infarct and cavitary lesions in the lungs, warranting inpatient admission (Figure 2A). The consolidative process observed on imaging was not further evaluated because the patient had undergone a bronchoscopic evaluation with biopsy at an outside hospital a week prior to presentation. This biopsy showed no malignancy or vasculitis, and bronchial brushings revealed benign bronchial epithelium with no reactive changes. Due to concern for septic emboli and toe discoloration, there was a high index of suspicion for infective endocarditis (IE). However, blood cultures, transthoracic echocardiography, and transesophageal echocardiography were negative for IE. To identify the source of the emboli, a positron emission tomography (PET) scan was performed, which showed increased activity in the nasal cavity (Figure 2C).

**Table 1:** Significant laboratory findings

Parameter	Patient values	Reference range
White blood cell count	21.59 k/µL	4.8–10.8 k/µL
Platelet count	749 k/µL	140–440 k/µL
Hemoglobin	12.4 g/dL	13.0–18.0 g/dL
C-reactive protein	154.7 mg/dL	<10.0 mg/dL
Erythrocyte sedimentation rate	92 mm/h	0–15 mm/h

**Figure 3:**
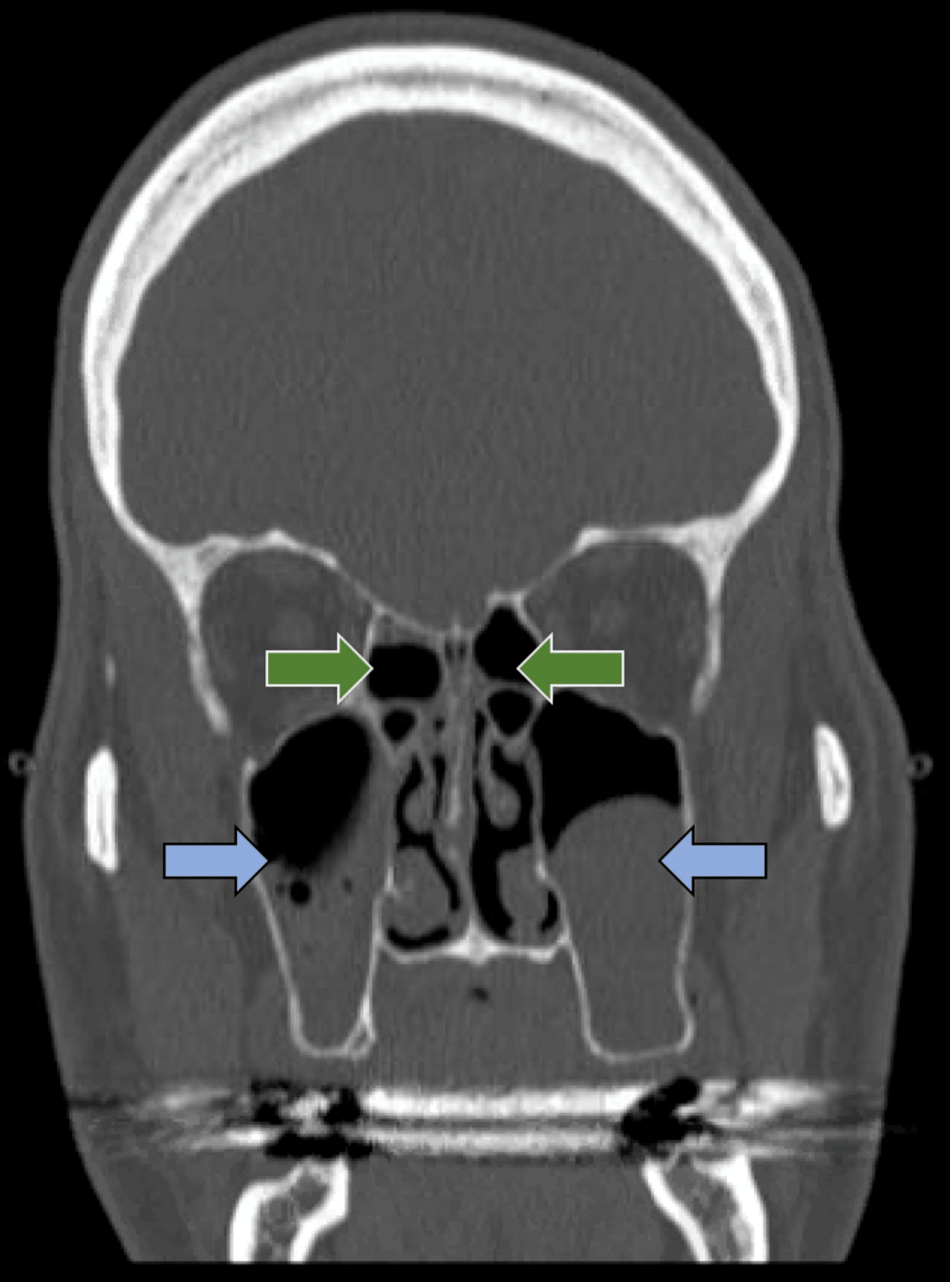
CT of sinuses CT of the sinuses shows scattered moderate mucosal thickening throughout bilateral ethmoid (green arrows) and maxillary sinuses (blue arrows) suggestive of chronic sinusitis.

A CT scan of the sinuses was then obtained for further evaluation, which showed findings consistent with sinusitis (Figure 3). An ear, nose, and throat (ENT) consultation was performed, and the patient subsequently underwent bilateral endoscopic sinus surgery with maxillary antrostomy and anterior ethmoidectomy. Intraoperative findings revealed a large septal perforation with crusting.

**Figure 4:**
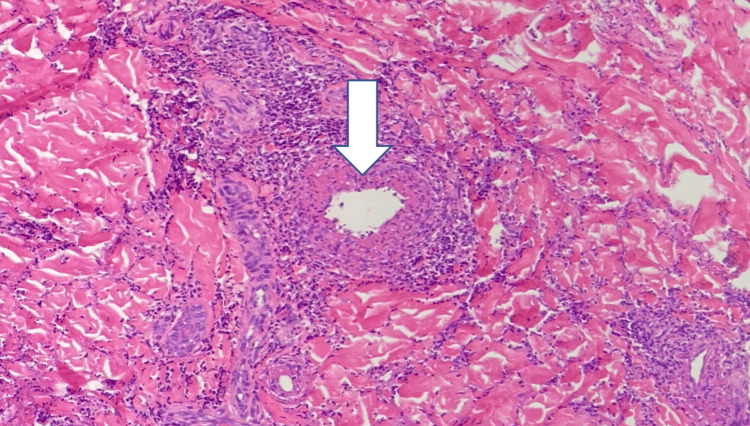
Skin biopsy findings showing leukocytoclastic vasculitis Biopsy findings showing central vessel with surrounding neutrophilic infiltration (arrow).

With no improvement from intravenous (IV) antibiotics, a punch biopsy of the right foot was performed, which showed findings consistent with leukocytoclastic vasculitis (Figure 4). Serology was positive for c-ANCA, with an elevated rheumatoid factor of 237 (0-14 IU/mL), high IgE of 1888 (≤214 kU/L), serine Proteinase 3 IgG of 644 (0-19 AU/mL), high Complement C1q of 87 (109-242 µg/mL), and Complement C3 of 181 (90-180 mg/dL). Based on these results, a diagnosis of GPA was made. Rheumatology was consulted, and the patient was started on high-dose methylprednisolone, 500 mg IV daily for three days, followed by 60 mg IV twice daily for maintenance. Although rituximab was recommended, due to financial constraints, methotrexate, 7.5 mg orally twice weekly was initiated following discussion with rheumatology. This eventually led to symptomatic improvement. The patient was advised to follow up with rheumatology, otorhinolaryngology, and pulmonology in the outpatient setting. He was also advised to receive the varicella (shingles) and pneumococcal vaccines. A follow-up chest CT in the outpatient setting showed stable findings.

## Discussion

The formation of granulomas in GPA begins with neutrophilic microabscesses, which ultimately result in complete or partial obstruction of blood vessels. Patients with GPA typically present with nonspecific symptoms, such as fever, generalized weakness, unintentional weight loss, and arthralgias. Sinusitis and epistaxis are the most common symptoms [3]. Prodromal symptoms may persist for weeks to months [4]. Involvement of the airways or pulmonary parenchyma in GPA leads to a range of symptoms, including cough, dyspnea, stridor, hemoptysis, and pleuritic pain [5-7]. Findings on chest imaging can vary, presenting as nodules, opacities that may be patchy or diffuse, and pulmonary infiltrates with hilar lymphadenopathy.

Skin involvement may be the initial clinical presentation in about 10% of cases, usually indicating active systemic disease [8,9]. Common skin lesions in GPA include palpable purpura, necrotic lesions, and ulcers, often involving the oral cavity. The lower extremities are frequently affected [8,9]. These findings may also be observed in IE, making it challenging to distinguish between GPA and IE, as noted by Rachdi et al. [10]. A positive c-ANCA is highly associated with GPA, with a sensitivity and specificity of over 90%. Histopathologically, leukocytoclastic vasculitis is seen, as observed in our case [11,12]. Blood cultures are crucial for differentiating GPA from IE, as they are positive in 90% of IE cases [10]. It is essential to distinguish GPA from IE because the immunosuppressive agents used to treat GPA can exacerbate infections and have life-threatening consequences [13,14].

If untreated, the mean survival duration for adults with GPA is approximately five months, with up to 80% of patients dying within a year and 90% within the second year [15]. Acute renal disease or hemoptysis is the most common cause of death. However, long-term survival has become possible with the use of prednisone and cyclophosphamide. Prompt diagnosis is therefore crucial. Most patients who receive corticosteroids and immunosuppressive agents have a favorable prognosis.

## Conclusions

GPA and IE are diseases affecting the vasculature, and they can have overlapping signs and symptoms, making differentiation challenging for clinicians. Cases that do not respond to traditional treatment for one of these conditions should prompt clinicians to consider an alternate diagnosis, as illustrated in our case. While IE tends to worsen with immunosuppression, GPA generally responds favorably to it.
